# Feasibility and usefulness of postoperative mobilization goals in the enhanced recovery after surgery (ERAS^®^) clinical pathway for elective colorectal surgery

**DOI:** 10.1007/s00423-024-03442-5

**Published:** 2024-08-31

**Authors:** Rico Wiesenberger, Julian Müller, Mario Kaufmann, Christel Weiß, David Ghezel-Ahmadi, Julia Hardt, Christoph Reissfelder, Florian Herrle

**Affiliations:** 1 Department of Surgery, Universitätsmedizin Mannheim, Medical Faculty Mannheim, Heidelberg University, Theodor-Kutzer-Ufer 1-3, 68167 Mannheim, Germany; 2 Institute for Medical Statistics, Universitätsmedizin Mannheim, Medical Faculty Mannheim, Heidelberg University, Theodor-Kutzer-Ufer 1-3, 68167 Mannheim, Germany; 3 Department of Anaesthesiology and Critical Care Medicine, Universitätsmedizin Mannheim, Medical Faculty Mannheim, Heidelberg University, Theodor-Kutzer-Ufer 1-3, 68167 Mannheim, Germany; 4 DKFZ-Hector Cancer Institute, University Medical Center Mannheim, Mannheim, Germany

**Keywords:** Minimal-invasive surgery, daVinci, Movisens, Garmin vivosmart, Motion sensors, Goal setting

## Abstract

**Purpose:**

Despite mobilization is highly recommended in the ERAS^®^ colorectal guideline, studies suggest that more than half of patients don’t reach the daily goal of 360 min out of bed. However, data used to quantify mobilization are predominantly based on self-assessments, for which the accuracy is uncertain. This study aims to accurately measure postoperative mobilization in ERAS^®^-patients by validated motion data from body sensors.

**Methods:**

ERAS^®^-patients with elective bowel resections were eligible. Self-assessments and motion sensors (movisens: ECG-Move 4 and Move 4; Garmin: Vivosmart4) were used to record mobilization parameter from surgery to postoperative day 3 (POD3): Time out of bed, time on feet and step count.

**Results:**

97 patients were screened and 60 included for study participation. Self-assessment showed a median out of bed duration of 215 min/day (POD1: 135 min, POD2: 225 min, POD3: 225 min). The goal of 360 min was achieved by 16.67% at POD1, 21.28% at POD2 and 20.45% at POD3. Median time on feet objectively measured by Move 4 was 109 min/day. During self-assessment, patients significantly underestimated their “time on feet”-duration with 85 min/day (*p* = 0.008). Median number of steps was 933/day (Move 4).

**Conclusion:**

This study confirmed with objectively supported data, that most patients don’t reach the daily mobilization goal of 360 min despite being treated by an ERAS^®^-pathway with ERAS^®^-nurse. Even considering an empirically approximated underestimation, the ERAS^®^-target isn’t achieved by more than 75% of patients. Therefore, we propose an adjustment of the general ERAS^®^-goals into more patient-centered, individualized and achievable goals.

**Registration:**

This study is part of the MINT-ERAS-project and was registered prospectively in the German Clinical Trials Register on 25.02.2022. Trial registration number is “DRKS00027863”.

## Introduction

Enhanced Recovery after Surgery (ERAS^®^) is an evidence-based and multimodal perioperative treatment strategy to improve the recovery process after surgery [1, 2]. In colorectal surgery, compliant ERAS^®^-treatment can achieve a halved postoperative complication rate [3] and a shortened hospital stay of ~ 2.5 days [4]. Early postoperative mobilization is one of the core components in the ERAS^®^ guidelines for colorectal surgery [5, 6]. Comprehensive mobilization is an independent predictor for reduced moderate to severe complications [3] and a reduction of inpatient length of stay [7]. The most recent duration-specific ERAS^®^ mobilization goals were published in the 3rd updated guidelines from 2013: a cumulative duration of 2 h out of bed on the day of surgery and 6 h from post-op day 1 (POD1) [8, 9]. In the latest 4th updated ERAS^®^ guidelines from 2018 for colorectal surgery, mobilization is still declared with a “strong” recommendation-level, but without specification regarding the extent [6]. The ERAS^®^ Interactive Audit System (EIAS) still uses the compliance target of 2 h on the day of surgery and 6 h from POD1 to assess protocol adherence [10, 11]. Studies have shown that mobilization is the ERAS^®^ component with the poorest adherence [12, 13]. Grass et al. showed that 58% of 1170 ERAS^®^ patients did not reach the 360 min out of bed on POD1 [14], in the study of Gustafsson et al. (*n* = 664) it was even higher at 72.5% [15]. Therefore, it can be assumed that at least half of the ERAS^®^ patients did not reach the 360 min out of bed daily target.

There is evidence in goal-setting research that unrealistically high goals can be counterproductive [16–18]. Höpfner and Keith showed experimentally that failure to achieve an excessively high target was associated with a decrease in self-confidence and motivation [19]. This study therefore takes a critical look at whether the general ERAS^®^ mobilization target is useful for the majority of patients.

However, the fact that the latest 4th ERAS^®^ guidelines avoid specifying targets is also problematic, because there is strong evidence that specific targets positively influence patient behavior [17, 20]. In addition, the lack of specific mobilization-instructions in ERAS^®^ guidelines contributes to clinicians interpreting “early mobilization” differently [21]. Hence, the mobilization target of the EIAS database (360 min out of bed/day) is not used consistently in the ERAS^®^ literature [22]. For example, some ERAS^®^-studies used to assess adherence the ability to walk at POD1 [12], active mobilization on POD1 [23] or successful mobilization to a chair within 12 h after surgery [3].

A general problem in assessing mobilization adherence is that data are predominantly based on subjective assessments by nurses or patients [24]. Since no objective data were collected, it is unclear how accurate subjective assessments really are.

Hence, in the present study evaluation we focused on accurate, multi-sensor patient activity assessment to analyze postoperative mobilization more objectively. Finally, those data should allow to critically discuss the feasibility and patient-relevance of the ambitious ERAS^®^ postoperative mobilization goal of 360 min/day.

## Material & methods

### Participants

Patients undergoing elective bowel surgery at the University Hospital Mannheim were eligible.

The inclusion criteria were ≥ 18 years, cognitive abilities to understand the study and informed consent, consent to treatment according to the ERAS^®^ protocol, Barthel index ≥ 10 points for mobility (assisted walking > 50 m). Reasons for exclusion were inability to have a fluent conversation (language barrier, hearing problems, mental states), previous study participation, PEG or parenteral nutrition, cardiac devices and expected incompliance concerning the protocol requirements (especially handling with technical devices).

A sample size of 50 patients was planned for this pilot-study design.

### Study design & procedure

This study evaluation is part of the MINT-ERAS^®^ project, a monocenter pilot-RCT project investigating the effect of a specific patient conversation style (Motivational Interviewing/MI [25]) on postoperative ERAS^®^ mobilization and nutrition goals. The results regarding the impact of MI on early mobilization are published in a separate detailed report [26]. In the present study evaluation – focusing on the overall mobilization data of ERAS^®^-patients recorded by three different body-motion sensors – data from both the intervention and the control group were analyzed together as a single-arm study.

The subjects were treated according to the certified ERAS^®^-pathway for colorectal surgery at the University Hospital Mannheim [27]. Prior to surgery, patients were educated as usually by the ERAS^®^-nurse on the objectives and process of ERAS^®^. The ERAS^®^-nurse is specifically responsible for the ERAS^®^-patients and cared for them daily after surgery (excluding weekends).

After study enrollment, participants were instructed on handling the technical equipment a few days before surgery (usually at the anesthesiologist appointment), and ERAS^®^ objectives were revisited. At the daily postoperative visits from POD0 to POD3 (usually between 6:00 pm and 8:30 pm, see Table 1), the sensors were checked and general data were asked (pain, nausea, activity, etc.). At the following day’s visit, data from the previous evening were added (e.g., if a patient was still out of bed for 30 min after the visit at POD1, it was recorded at the POD2 visit). At POD0, the three motion sensors were attached to the study participants, and at POD3 after 8 pm, they were removed. A telephone follow-up was conducted 28–32 days after surgery to complete questionnaires.

**Table 1:** Study visits on top to the regular ERAS^®^-nurse consultations

Visit	1	2	3	4	5	6
**Day**	few days before op	day 0 (op)	day 1	day 2	day 3	*day 28*–*32 after op*
∼** time expenditure [min]**	20	15				*10*
**location**	Outpatient clinic	clinic				*phone*

### Measuring devices

For objective activity profiling, three motion sensors (Garmin Vivosmart 4, Move 4 and ECG Move 4) were used and recorded data from 11:59 pm on day 0 for 68 h until 8:00 pm on POD3.

**Fig. 1:**
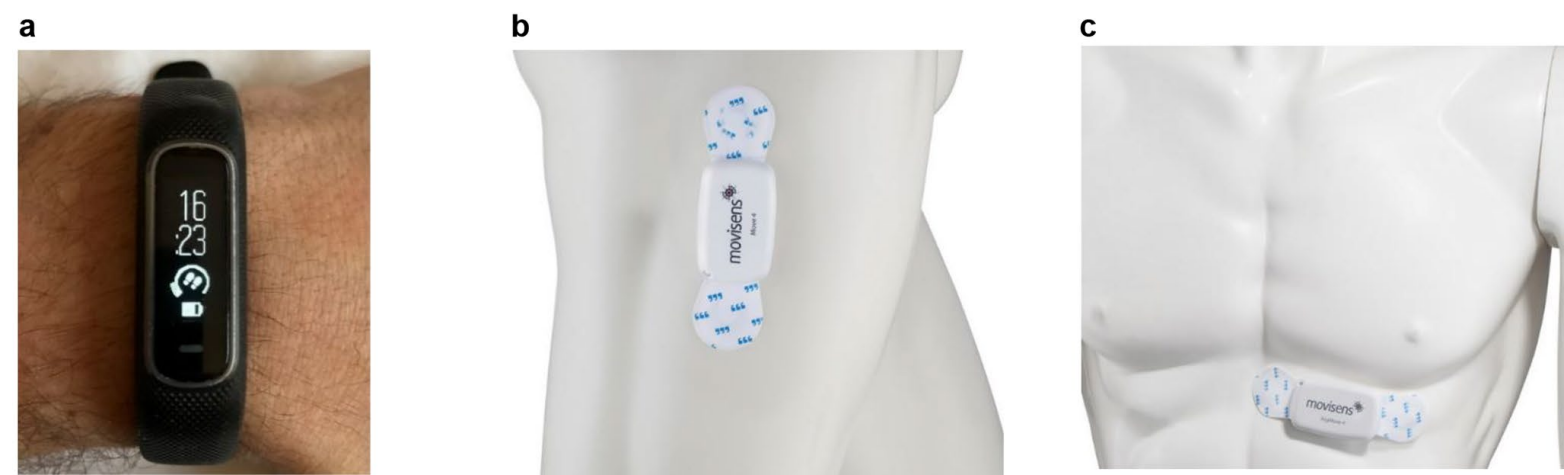
(**a**) Garmin Vivosmart 4 (**b**) Move 4 (**c**) EKG Move 4

The commercial tool Garmin Vivosmart 4 (GVS4) [28] was worn on the wrist (see Fig. 1a). The GVS4 is waterproof, has a battery life of up to 7 days, and weighs between 16.5 g and 17.1 g. For analyzing step count, the GVS 4 was synchronized via Bluetooth with the “Garmin Connect” app [29] of a study smartphone.

The Move 4 [30] and EKG Move 4 [31] activity sensors were developed by movisens GmbH (Karlsruhe, Germany) and have been validated concerning step count (mean percentage deviation 0,6%) [32] and activity classes (accuracy 98,2%) [33] for scientific research purposes. The sensors are waterproof, the battery life is at least 3 days, they have a size of 62.3 mm x 38.6 mm x 11.5 mm and weigh 25–26 g. During the measurement, integrated sensors (3D acceleration, rotation, pressure, temperature) continuously record raw physical data, the ECG Move 4 also includes an ECG sensor. The configuration of the sensors was done by the software “Sensor Manager“ [34]. The sensors were attached with adhesive electrodes, the Move 4 vertically on the lateral right thigh (see Fig. 1b), the ECG Move 4 horizontally from the xiphoid to the underside of the left chest (see Fig. 1c). Body hair was shaved beforehand. The electrodes were checked for tightness during the daily visit and replaced if necessary. The “Data Analyzer” calculates physiological parameters such as activity classes (lying, sitting, standing, walking), number of steps, and cardiac parameters (heart rate, heart rate variability) from the raw data and presents them in analysis overviews [35]. To register steps and the activity class “walking”, at least three consecutive steps must be taken (time to take one step 0,2–2 s each) [36].

### Endpoints

To measure mobilization, the ERAS^®^ compliance target “time out of bed” POD0–POD3 (patient self-assessment) was recorded. To specify the activity profile and simultaneously support it with objective values, the number of steps (objective by Move 4, ECG Move 4 & GVS4) and the time on feet (subjective and objective by Move 4, sum of activity classes standing + walking) of POD1–POD3 were measured. By measuring “time on feet” subjectively as well as objectively, it was intended to draw conclusions about the accuracy of self-assessments by calculating the deviations. All data on POD3 refer to the period from 0:00 a.m. – 8:00 p.m. and all data on POD0 refer to the period after the end of surgery.

Further endpoints were mobilization at POD0 (arrival time on ward, mobilized yes/no, duration until first mobilization), metabolic equivalent (POD1–POD3), pain (maximum/average) as well as complications (POD0–POD3) and duration of hospital stay. Questionnaires were used to assess activity before surgery (IPAQ - International Physical Activity Score [37], pre-op), patient satisfaction (PEQ – Patient Experience Questionnaire [38], POD28–32), activity of daily living (Barthel-Index [39], pre-op und POD3) and Quality of Recovery (QoR-15 – Quality of Recovery [40], pre-op, POD 1/3/28–32). Minimum and maximum achievable scores are PEQ (0–100), Barthel (0–100), QoR-15 (0–150). In addition, correlation between postoperative “time on feet” (Move 4) and preoperative activity (IPAQ) or age was tested.

### Statistical analysis

Only complete data sets were included for the statistical analysis of the individual days. For example, if patients were already discharged on POD3, they were included in the analyses from op-day to POD2, but not on POD3.

Continuous data are presented as median [quartile range = Q1-Q3] [range = minimum-maximum] and/or arithmetic mean ± standard deviation. For comparison of metric-scaled characteristics “time on feet” subjective & objective, Wilcoxon test for paired samples was used. Correlations were presented using Spearman’s rank correlation coefficient. A *p*-value < 0.05 (two-sided test) is considered statistically significant. SAS^®^ software (SAS Institute, Inc., USA) was used for statistical analyses.

## Results

### Personal data & sample

Between March 2022 and July 2022 97 patients were screened. 60 patients participated in the final study. Originally, only 50 study participants were planned as pilot study sample size, but due to some incomplete data sets, the sample size was increased to 60 during the recruitment period (approved by the ethics committee). According to intention-to-treat principle all 60 randomized study participants were included in the statistical analyses. The following deviations exist: The self-assessment “time out of bed” was collected from POD3 onwards by subjects 11 & 12. Reduced samples on individual days are due to one death on POD2 and five discharges on POD3. In addition, during the step count by GVS4 at POD3, the battery ran out for two subjects and once the fitness wristband was not worn continuously. Since the Move 4 fell of a few times during the initial phase (without data loss) when putting on / taking off pants, it was additionally covered with an adhesive film. Apart from this, all study participants were compliant with the protocol requirements and wore the motion sensors throughout day and night.

The study participants had an average age of 60.7 ± 13.3 years (range = 30–82 y.), BMI of 26.4 ± 5, and 56.67% were male (see Table 2). 75% of the subjects were classified ASA II, 21.67% ASA III, and 3.33% ASA I. The mean duration of surgery was 216.7 ± 110.8 min, and 35% of the patients received a stoma during surgery.

63.33% of procedures were minimal-invasive (laparoscopic or daVinci), 33.33% were primarily open, and 3.33% were converted (see Table 2). The two most common surgical indications were malignant neoplasms (46.67%) and stoma-reversal / restoration of bowel continuity after Hartmann’s Procedure (35%). The study included a wide range of bowel surgical procedures (see Table 2). Apart from one procedure (pelvic tumor debulking after rectal carcinoma), bowel resection was performed in all other operations.

**Table 2:** Baseline characteristics & su﻿rgery of study participants

Gender (m/f)	34/26	**Op-indication**, **n [%]**		
Age in years	62.2 ± 13.8	Malignant disease		28 [46.67%]
BMI	25.6 ± 4.6	Crohn/ulcerative colitis		2 [3.33%]
ASA (I/II/III)	2/45/13	Diverticulitis		3 [5%]
Duration of surgery in min	216.7 ± 110.8	Stoma-Rev.&Hartmann		21 [35%]
Stoma received, n [%]	21 [35%]	Rectal prolaps		3 [5%]
Blood loss during op in ml	115 ± 181.3	Others		3 [5%]
Complications during op, n [%]	1 [1.67%]	**Type of surgery**,** n [%]**		
Pre-op bowel preparation, n [%]	36 [60%]	Colonic resection		12 [20%]
Pre-op anemia, n [%]	20 [33.33%]	Rectal resection		19 [31.67%]
Diabetes, n [%]	7 [11.67%]	Stoma revision		21 [35%]
Alcohol abuse, n [%]	7 [11.67%]	Others		8 [13.33%]
Smoking, n [%]	11 [18.33%]	**Type of intervention**,** n [%]**		
Pre-op immunosuppression, n [%]	3 [5%]	primary open		20 [33.33%]
Pre-op chemotherapy, n [%]	6 [10%]	converted		2 [3.33%]
EKOG grade (0/1)	30/30	laparoskopic		24 [40%]
Walking aid before surgery n [%]	2 [3.33%]	robotic assisted (daVinci)		14 [23.33%]

### Time out of bed POD1 – POD3 (Table 3)

Across POD1 to POD3, study participants spent a median of 215 min/day out of bed. At POD1, the median mobilization time was 135 min; at POD2 and POD3, it increased to 225 min. The ERAS^®^ compliance target of 360 min was reached by less than 25% on all three days (POD1: 16.67%, POD2: 21.28%, POD3: 20.45%).

**Table 3:** Time out of bed (self-assessment in minutes)

	Median [Q1-Q3]	[Min-Max]	Mean ± SD			
POD1 (*n* = 48)	135 [35–240]	[0-930]	184.8 ± 201.7			
POD2 (*n* = 47)	225 [90–310]	[10–890]	241.8 ± 184			
POD3 (*n* = 44)	225 [120–285]	[15–855]	240.2 ± 181.1			
⌀ per POD (*n* = 50)	215 [131.7–280]	[12.3-891.7]	223.4 ± 170			

*POD* = postoperative day, *min* = minimum, *max* = maximum, *SD* = standard deviation

### Time on feet (self-assessment & objective; Table 4)

**Table 4:** Time on feet (standing + walking)

	Objective (Move 4) Median [Q1-Q3] [Min-Max]		Self-assessment Median [Q1-Q3] [Min-Max]	*p*-value*
POD1 (*n* = 60)		91 [26.5–152] [0-334]	60 [20–120] [0-660]	0.016
POD2 (*n* = 59)		130 [54–198] [17–443]	90 [60–170] [10–480]	0.01
POD3 (*n* = 54)		122 [79–200] [6-589]	120 [45–180] [8-660]	0.083
∑ POD1-3 (*n* = 54)		310 [175–521] [34-1362]	255 [150–422] [28-1560]	0.01
⌀ / day (*n* = 60)		109 [58.5-175.5] [10–454]	85 [52.5-148.7] [9.3–520]	0.008

* Wilcoxon test for paired samples. *POD* = postoperative day, *min* = minimum, *max* = maximum

Objectively measured by Move 4, study participants were median 109 min/day (POD 1–3) on their feet (standing and walking), compared with only 85 min/day in the self-assessment. Participants significantly underestimated themselves (⌀ / day, *p* < 0.008). Across POD1–3, the median deviation in self-assessment compared with the objective score was − 17.3 min/day (≙–15.87% of 109 min/day).

**Table 5:** Step count

			Median [Q1-Q3]	[Min-Max]	Mean ± SD
Move 4		POD1 (*n* = 60)	526 [49-2185]	[0-17565]	1478 ± 2659
		POD2 (*n* = 59)	1209 [246–3372]	[0-19808]	2198 ± 2987
		POD3 (*n* = 54)	1212 [548–3051]	[0-15259]	2255 ± 2840
		∑ POD1-3 (*n* = 54)	2566 [1022–8923]	[0-52632]	5747 ± 8284
		⌀ per POD (*n* = 60)	933 [376–3026]	[0-17544]	1972 ± 2685
EKG Move 4		POD1 (*n* = 60)	304 [9-2010]	[0-17986]	1435 ± 2725
		POD2 (*n* = 59)	1288 [117–3345]	[0-19545]	2184 ± 3061
		POD3 (*n* = 54)	1244 [580–3618]	[0-16048]	2404 ± 3240
		∑ POD1-3 (*n* = 54)	2758 [801–8243]	[0-52501]	5884 ± 8677
		⌀ per POD (*n* = 60)	964 [276–2820]	[0-17500]	1990 ± 2794
GVS 4		POD1 (*n* = 60)	1114 [308–3149]	[9-19578]	2189 ± 3131
		POD2 (*n* = 59)	2380 [721–4917]	[13-21308]	3095 ± 3439
		POD3 (*n* = 51)	1995 [1066–4141]	[26-21557]	3318 ± 3851
		∑ POD1-3 (*n* = 51)	4420 [2603–12192]	[48-57188]	8283 ± 10,022
		⌀ per POD (*n* = 60)	1800 [875–4390]	[16-19063]	2854 ± 3225

*GVS4* = Garmin Vivosmart 4, *POD* = postoperative day, *min* = minimum, *max* = maximum, *SD* = standard deviation. Step count rounded to whole steps

### Step count (Table 5)

The motion sensors measured a median of 933 steps/day (Move 4), 964 steps/day (ECG Move 4), and 1800 steps/day (GVS4) across the period of POD1 to POD3. The GVS4 collected a higher number of steps than the two movisens sensors on all three days. The data of all three sensors are right-skewed distributed, recognizable by the higher mean values (compared to the median), the smaller distance of the median to Q1 than to Q3, and the high maxima (up to 21557 steps for GVS4 at POD3). The cumulative range (POD1–POD3) measured by Move 4 is 0–52632 steps.

### Mobilization POD0

The median [Q1-Q3] time point at which the subjects arrived on the ward from the recovery room was 5:10 pm [2:28pm-7:05pm], minimum was 11:15 am and maximum 10:31 pm. 33 of the 60 subjects were mobilized to their feet on the day of surgery. Of the 33 subjects who were mobilized, a median [Q1-Q3] of 15 min [5–30] was spent out of bed on POD0, the maximum was 120 min. Based on all 60 study participants, the median [Q1-Q3] duration from the end of surgery (suture) to the first mobilization was 430 min [261–966]; only one person was mobilized for the first time on POD2 (after 2232 min).

### Other outcome-variables (Tables 6 and 7)

On median, subjects had a MET level < 1.5 (inactive) at POD1 98.65% of the time ( ≙ 23 h + 19.5 min), then through POD2 (96.46%) and POD3 (95.54%) the “inactive” proportion of time decreased continuously (see Table 6). 65% of the subjects achieved the activity class “high” in the IPAQ, 30% “medium” and only 5% “low” (see Table 7). Absolute MET level in the IPAQ (*r* = − 0.133, *p* = 0.339) and age (r = + 0.251, *p* = 0.067) showed no significant relationship with cumulative (POD1–POD3) time on feet. In the questionnaire “Quality of Recovery”, the lowest median was reached after surgery at POD1 (106.5) and then increased through POD3 (114) to the highest value on POD28–32 (131), which was higher than the baseline preoperatively (128). Postoperative complications occurred once at POD0 (1x Clavien-Dindo (CD) Class I), three times at POD1 (2x CD I, 1x CD III), twice at POD2 (1x CD I, 1x CD V), and twice at POD3 (2x CD II). The median [Q1-Q3] regarding the patient-reported maximum pain peak (scale 0–10) was 5 [3–8] at POD0, 6.5 [4–8] at POD1, 4 [3–6] at POD2, and 4 [2–6] at POD3. Regarding the pain average over the whole day, the median [Q1-Q3] at POD0 was 4 [2–5] at POD1 3 [2.5-5], at POD2 3 [1–4], and at POD3 2 [1–4]. Postoperative hospital stay had a median of 5 days [Q1-Q3: 4–7; range: 3–20].

**Table 6:** Metabo﻿lic equivalent (MET-level) / activity level measured by EKG Move 4 (in %)

	MET-level	Median [Q1-Q3]	[Min-Max]		Mean ± SD
POD1 (*n* = 60)	inactive < 1,5	98.65 [95.63–99.72]	[83.96–100]	97.19 ± 3.49	
	low 1,5–3	1.25 [0.28–3.13]	[0.00-8.26]	1.97 ± 2.1	
	medium 3–6	0.07 [0.00-1.22]	[0.00-1.60]	0.83 ± 1.89	
	high > 6	0.00 [0.00–0.00]	[0.00-0.21]	0.01 ± 0.03	
POD2 (*n* = 59)	inactive < 1,5	96.46 [93.47–99.51]	[82.99–100]	95.94 ± 3.82	
	low 1,5–3	2.43 [0.49–4.31]	[0.00-9.44]	2.87 ± 2.5	
	medium 3–6	0.49 [0.00-1.6]	[0.00-13.82]	1.19 ± 2.06	
	high > 6	0.00 [0.00–0.00]	[0.00-0.21]	0.00 ± 0.03	
POD3 (*n* = 54)	inactive < 1,5	95.54 [93.08–97.83]	[66.06–99.92]	94.54 ± 5.59	
	low 1,5–3	3.42 [1.92–5.25]	[0.08–15.10]	3.84 ± 3.13	
	medium 3–6	0.50 [0.00-2.25]	[0.00-18.85]	1.61 ± 3.15	
	high > 6	0.00 [0.00–0.00]	[0.00-0.17]	0.01 ± 0.03	

Percentage in a MET-Level of 24 h (POD1 / POD2) and 20 h (POD3). *POD* = postoperative day, *min* = minimum, *max* = maximum, *SD* = standard deviation

**Table 7:** Questionnaires

IPAQ rank, pre-op, high/medium/low, *n* [%]	39 [65%] / 18 [30%] / 3 [5%]
IPAQ absolute, pre-op, Median [Q1-Q3]	4265 [2226–7005]
Barthel-Index, pre-op / POD3, Median [Q1-Q3]	100 [90–100] / 95 [90–100]
QoR-15, pre-op, Median [Q1-Q3]	128 [118-140.5]
QoR-15, POD1 / POD3 / POD30, Median [Q1-Q3]	106.5 [91-126.5] / 114 [93–130] / 131 [117–140]
PEQ, POD30, Median [Q1-Q3]	
physicians / nursing / hospital	90 [75–100] / 85 [80–100] / 80 [68–88]

*IPAQ* = International Physical Activity Score (activity before surgery), *Barthel-Index =* activities of daily living, *QoR-15* = Quality of Recovery 15, *PEQ* = Patient Experience Questionnaire (patient satisfaction), *POD* = postoperative day

## Discussion

Several previous studies have shown that a high percentage of patients fail to achieve the ambitious ERAS^®^ compliance goal of 360 min/day out of bed [3, 12, 14, 15]. However, since the previous data were collected by subjective assessments, the accuracy of these data is unclear so far. To measure mobilization as accurately as possible in this study, we recorded activity with three different body sensors and compared it with self-assessment. With the data from the present study, we could show that less than a quarter of ERAS^®^-patients achieved the ERAS^®^ compliance goal of 360 min/day out of bed. The median time out of bed across the first three postoperative days was 215 min/day, more than 2 h away from the general ERAS^®^ mobilization target.

Within the ERAS^®^-program at the Universitätsmedizin Mannheim, there are specially trained physiotherapists and an ERAS^®^-nurse; additionally, all nursing staff on the ward have undergone ERAS^®^-training. Patients receive a detailed informational talk and an ERAS^®^-brochure preoperatively, which also include patient requirements (among other things to be 360 min out of bed from POD1). Furthermore, it should be considered that fitness wristbands can increase physical activity in hospitalized patients [41], especially when visual feedback is provided via a display [24]. Since we used fitness wristbands providing feedback in our study, it can be assumed that mobilization in this work tended to be even higher than in the broad range of ERAS^®^-treatments without study conditions. So, the patients had all the important information, the multidisciplinary staff was ERAS^®^ trained and all patients were additionally equipped with mobilization-enhancing fitness wristbands.

Despite this described setting, we could show with objectively supported data that only about 20% achieved the ERAS^®^ compliance target of 360 min out of bed. Objectively supported means that the accuracy of patient self-assessments has been checked with motion sensors. By recording “time on feet” (standing & walking) by self-assessment as well as objectively by the sensors, the accuracy of subjective data could be approximated in the present study. Due to the systematic underestimation (at all 3 PODs) of the parameter “time on feet”, it can be assumed that patients also underestimated themselves by about 15.87% for “time out of bed”. However, if 15.87% is added to Q3 of each POD1–3, the third quartile remains below 360 min at all three PODs. Thus, even taking underestimation into account, less than 25% met the ERAS^®^ compliance target.

In our study, a mobilization adherence (≥ 360 min/day) of 16.67% was achieved on POD1. Compared with other studies, adherence was between 23.5% and 27.5% observed by Gustafsson et al. [15]. , 43,9% observed by Schwenk et al. [13]. and 42% observed by Grass et al. [14]. Adherence in Grass et al. increased to 58% at POD2 (vs. 21.28% in our study) and even to 69% at POD3 (vs. 20.45% in our study, cave: only until 8:00 pm). The higher values of Grass et al. might be surprising considering that in our study (in contrast to Grass et al.) the above-mentioned mobilization-promoting measures were applied. However, the 1170 records in the study of Grass et al. were analyzed retrospectively and are based only on subjective assessments of the nursing staff. In addition, when comparing individual monocentric studies, the workflow may vary clearly between the clinics.

55% of the subjects got up for the first time on the day of the operation, which is similar to the values from other studies with 45% [14], 48.39% [3], 54.5% [42] and 59,6% [13]. Subjects mobilized at POD0 spent a median of 15 min out of bed, with only one person reaching the ERAS^®^ compliance target of 120 min. A main reason for the lack of mobilization at POD0 is probably the end of surgery respectively the time at which the patients arrive on the ward. With a median time point of 5:10 pm, more than half of the patients arrive on the ward from the recovery room after the usual working hours of the ERAS^®^-nurse and physiotherapists. Nevertheless, it should be noted, that despite poor adherence regarding the ERAS^®^ mobilization goals, one of the main goals of ERAS^®^ (early discharge) was successfully achieved in our study with a median postoperative hospital stay of 5 days.

Even though the ERAS^®^ compliance target of the EIAS database only refers to “time out of bed”, it can be assumed that components of moderate activity on feet (standing & walking) are superior to sitting only [9]. For example, the number of steps has been shown to correlate negatively with length of hospital stay [24] and complications [43]. Time on feet (standing & walking) between POD1–POD3 in our study was 109 min/day (objective), comparatively higher than in an abdominal surgery study (*n* = 118; neoplasms colorectal, ovarian, or urinary bladder) with objective values ranging from 42 min (control) to 78 min (intervention) per day [44].

It is striking that the commercial tracker GVS 4 counted considerably more steps than the two scientific research sensors from Movisens (∑ POD1–3: 4420 vs. 2758/2566). The reason for this are programming rules in movisens: steps are only counted when three consecutive steps are taken [36]. This minimizes noise and avoids counting “false steps”; however, in the postoperative setting, it can also cause that very “careful steps” (slow, small, isolated) are not registered adequately. The GVS4 is more sensitive, but in return the noise is much higher. For example, GVS4 already registered a few steps of participants, even though they had not yet stood up at that time.

In this study, unfortunately, it was not possible to calculate the time out of bed completely objectively (by the sum of the activity classes sitting, standing and walking) for two reasons: Firstly, the ECG Move 4 registers the activity class “sitting” at a horizontal upper body angle of > 30 degrees, and therefore periods with the backrest raised in bed were also registered as “sitting” (e.g. for reading, with gastric tube, sometimes also during sleeping, etc.). Increasing the angle for registering “sitting” is also problematic, since in that case patients sitting slightly at an angle could be registered as lying down. Second, even with adequate “sitting” registration, it is not possible to distinguish whether a patient is really sitting outside the bed (ERAS^®^-target) or merely at the edge of the bed. For these reasons, self-assessment of “time out of bed” was recorded from the eleventh study participant onwards, which was the main reason for a reduced sample size together with early discharges on POD3. Flavio Fiore et al. measured time out of bed both by the ActiGraph (sitting + standing + walking) and by self-assessment [42]. In the results, the objective measurement by the ActiGraph is up to 100% higher in median than the self-assessment; here the authors also discussed an incorrect classification of activity classes due to an elevated backrest. There are two ideas on how time out of bed could be measured completely objectively in future studies. The first idea is a technical localization of the participants, so that time periods outside a pre-programmed area around the patient’s bed are measured as “time out of bed”. The second idea are pressure-sensitive overlays for the bed, which can record periods without weight influence (threshold e.g. at 15 kg, so that bedspread, books, bags, etc. are not registered).

In summary, the data from this study, in comparison to the relevant literature, demonstrated that the current ERAS^®^ compliance target of the EIAS database (360 min out of bed) is not achieved by most patients. There is evidence that setting goals too high [16] and failure to achieve goals [19] have a negative impact on parameters such as self-confidence and motivation, which can negatively affect performance [18, 45]. It is noteworthy that the current ERAS^®^-guidelines of colorectal surgery do not specify daily mobilization goals at all. This may lead to inconsistent implementation between hospitals [21, 22]. Additionally, specific and achievable goals have positive effects on patient engagement [20]. It was shown, with a medium effect size (d = 0.42 to d = 0.56), that more specific goals lead to better performance than no specification or “do your best” goals [17]. In the literature, the best results are achieved when goals are difficult but reachable [17, 18].

In this paper, we will propose individualization of daily ERAS^®^ mobilization goals as a possible effective strategy. Since in our study postoperative activity did not correlate significantly with the age or subjective preoperative activity (IPAQ), these parameters should only play a minor role in individualization. Interestingly, the positive correlation between “age” and “time on feet” just missed significance (*p* = 0.067), which means that in tendency older people were even slightly longer on feet than younger people. A meaningful individualization could be done by the ERAS^®^-nurse in cooperation with the patients in line with the shared decision making (SDM) approach [46]. In their review, Rose et al. provide an overview of the positive effects of SDM in goal setting in a rehabilitation context; increased patient satisfaction and motivation were identified as important mechanisms [47].

Considering our lessons learned, the individualization of the ERAS^®^ mobilization goal and subsequent patient motivation could proceed as follows: In the preoperative ERAS^®^ consultation, patients are informed that spending as much time as possible out of bed has a positive effect on the recovery process. Subsequently, patients should set their individual goal for POD1, which they personally consider realistic and also are willing to achieve. The ERAS^®^-nurse provides support and ensures that the goals are specific (in hours), not too low/high and documented (e.g. ERAS^®^-diary). Patients who struggle with the specification of goals could be informed that on average about 2.5 h (150 min) are achieved out of bed on POD1 (which is about the median of our study of 135 min). After the operation (in the evening at POD0 or in the morning at POD1), the preoperative goal set for POD1 should be discussed again (and adjusted if necessary). At POD2 early in the morning (or at POD 1 in the evening), the mobilization at POD1 should be evaluated and based on this, a new individual daily target for POD2 would be set via SDM. The new daily target should be higher than the achieved value of POD1. In this way, the principle is similar to the mechanism of fitness trackers, which adjust the target for the following day depending on the achievement of a daily target (e.g. steps) [48]. The same procedure can be used for the following postoperative days. Although a goal for surgery day would also be useful, it is difficult to assess due to the uncertain end of surgery and dependence on staff for initial mobilization. To avoid early failure, we recommend the general goal of “first time standing up” for surgery day (currently achieved by about 50% [3, 13, 14]). In addition to time out of bed, walking goals (e.g., walking down the ward x times) are also possible within the individualized goals. It should be empirically tested whether individualized and participatory ERAS^®^ mobilization goals lead to better mobilization than the general daily goal of 360 min or no specific goal at all (as in the current ERAS^®^ guidelines). With new research, the intention is to close the current evidence gap regarding the imprecise mobilization recommendation within the next update of the ERAS^®^ colorectal guideline.

## Conclusion

In this study, postoperative mobilization of ERAS^®^-patients was measured as accurately as possible using three different motion sensors. Thereby it could be demonstrated that only about 20% of patients achieved the ERAS^®^ compliance goal of 360 min/day out of bed. Evidence from goal setting research suggests that the best performance is achieved through specific goals that are challenging yet achievable. To provide optimal goal targets for as many patients as possible, this study proposes an adjustment of the general ERAS^®^ mobilization goals to individualized goals defined participatively between ERAS^®^ nurse and patient. In follow-up RCTs, it is needed to test whether individualized ERAS^®^ goal setting and motivation techniques lead to improved mobilization. Additionally, the new research findings should help to adapt and solidify the recommendations for mobilization in future updates of the ERAS^®^ guidelines.

## Data Availability

No datasets were generated or analysed during the current study.

## Declarations

### Publication history

The submitted manuscript has not yet been published. Only an abstract of the paper is available online as part of the ERAS World Congress (https://erascongress.com/program/2023/P8.cgi).
